# Effect of Plateau Pika Disturbance on Plant Aboveground Biomass of Alpine Meadows at Two Different Scales

**DOI:** 10.3390/plants11172266

**Published:** 2022-08-31

**Authors:** Xiaoxing Wei, Zhenggang Guo

**Affiliations:** The State Key Laboratory of Grassland Agro-Ecosystems, Key Laboratory of Grassland Livestock Industry Innovation, Ministry of Agriculture and Rural Affairs, College of Pastoral Agriculture Science and Technology, Lanzhou University, Lanzhou 730020, China

**Keywords:** alpine meadow, small burrowing herbivore, plant productivity, functional group, disturbance intensity

## Abstract

Disturbance by small burrowing herbivores often has an impact on plant aboveground biomass of grassland because it makes grasslands into a mosaic of discrete vegetated surfaces and bare soil patches. This study focuses on the plateau pika (*Ochotona curzoniae*) to investigate the effect of the disturbance by a small burrowing herbivore on plant aboveground biomass through upscaling the quadrat scale to the plot scale across five sites. This study showed that the plateau pika disturbance reduced sedge biomass and increased forb biomass. In contrast, they did not affect plant community biomass, grass biomass and legume biomass at the quadrat scale across the five sites. At the plot scale, that is, when the bare soil patches with a lack of plants were considered, plateau pika disturbance induced lower aboveground biomass of the plant community, sedge and legumes, while it had no relationship with grass biomass and forb biomass. As the disturbance intensity increased, the aboveground biomass of the plant community and sedge decreased, whereas the grass biomass showed a hump-shaped trend. These results indicate that plateau pika disturbance might be not beneficial to alpine meadows given the aboveground biomass of the plant community at the plot scale. In contrast, the intermediate disturbance intensity improves the grazing quality of alpine meadows through the higher grass biomass. The findings of this study imply that the plot scale is better than the quadrat scale to investigate the influence of the disturbance by a small burrowing herbivore on the plant aboveground biomass, and that management of a small burrowing herbivore needs to consider its disturbance intensity.

## 1. Introduction

Grassland, one of the most important vegetation types, covers 31–43% of the terrestrial area on Earth [1], and its sustainability is vital to providing persistent goods and services for sustaining flora, fauna, and human needs [2]. Plant productivity is considered an important modality by which to evaluate grassland sustainability [3]. Several studies have identified that the plant productivity of grassland is affected by many biotic factors, such as plant diversity [4], livestock [5], and small burrowing herbivores [6,7].

Small burrowing herbivores are common biotic factors in grasslands, and their disturbance can influence plant productivity in several ways. Disturbance by small burrowing herbivores decreases the plant productivity by directly consuming plants, clipping tall plants to facilitate predator detection [8,9], burying vegetation [6,10], or indirectly inducing bare soil patches with a lack of plants [7,11,12]. In contrast, the activities of small burrowing herbivores can also increase plant productivity due to increased soil nutrition concentration [10] and can stimulate compensatory plant growth [13]. In addition, they can also stimulate plant growth by removing senescent leaves that decrease light and soil water for younger and more active tissues [14].

Previous studies show that the disturbance by small burrowing herbivores can either increase [14,15], decrease [16,17,18], or have no effect on plant productivity [19,20], and this difference is likely due to the sampling methods. In most published literature, many studies have placed quadrats only on vegetated surfaces with and without small burrowing herbivores to sample plants [18,19,20,21], and other studies have randomly placed quadrat to sample plants [15,18,22]. These existing sampling methods often lead to uncertainty in examining the effect of the disturbance by small burrowing herbivores on plant productivity due to neglecting bare soil patches with a lack of plants, or ignoring the great heterogeneity caused by small burrowing herbivores [6,10,23]. Recently, a plot-scale method was applied to estimate the effect of the disturbance by a small burrowing herbivore on soil carbon and nitrogen stock [24,25]. Plant productivity at the plot scale, defined as plant productivity measured in vegetated surface adjusted for the lack of productivity in bare soil patches, may properly assess the response of plant productivity to the disturbance by small burrowing herbivores. However, little attention has been paid to its effect on plant productivity at the plot scale.

As a common rodent, plateau pika (*Ochotona curzoniae*), is a small burrowing herbivore with an average weight of 150 g native to grasslands in Asia [6,26], especially alpine meadows in the Qinghai-Tibetan Plateau [11,12,20,23,27]. Plateau pikas can produce 2–5 litters (litter size range = 2–7) at 3-week intervals between each litter in the breeding season of April to August [28], and these animals are social animals that live in family groups consisting of two to five adults and their young that do not disperse in their year of birth [29,30]. Plateau pikas show territorial behavior and their home range is 1262.5–2308 m^2^ [26]. Pikas are non-hibernators, and the range of activity is generally about 20 m away from the central burrow. This animal often develops alpine meadows into discrete mosaics of vegetated surface and bare soil patches [6,12] and shapes alpine meadows, even contributing to alpine meadow degradation because its population density can grow rapidly within a relatively short period and is the highest in August ranging from 100 to 400 individuals per hectare [30]. Consequently, the plateau pika is generally considered a pest in China [27,31], as well as the prairie dog (*Cynomys* spp.) in the USA during 1982–1992 [32]. However, some studies have argued that the plateau pika is a keystone species for the alpine meadow ecosystem in the Qinghai-Tibetan Plateau [31,33] because the plateau pika serves as food for many predators, and its burrows serve as breeding habitats for small birds and lizards [31]. In addition, an increasing number of studies verify that the plateau pika disturbance often increases soil nitrogen, carbon concentrations [23], and soil temperature [18], whereas it decreases soil water content [20]. These changes can induce potential growth changes in different functional groups of the plant community because they have different abilities to adapt to changing environments [34,35,36]. The growth changes in plant functional groups can, in turn, alter plant productivity of the community. Therefore, it is necessary to examine the impact of plateau pika disturbance on the plant productivity of four functional groups (*Cyperaceae*, *Gramineae*, *Leguminosae*, *Forb*) and community at both quadrat and plot scales, which can not only present a pattern of the effect of the disturbance by a small burrowing herbivore on plant productivity, but also help develop insight into the role of plateau pikas in the alpine meadow ecosystems of the Qinghai-Tibetan Plateau.

This study investigates the effect of plateau pika disturbance by upscaling the measurement of plant aboveground biomass from the quadrat scale to the plot scale across multiple sites, and hypothesizes that (1) plateau pika disturbance does not directly change the plant productivity of the community at the quadrat scale; (2) plateau pika disturbance does decrease the plant productivity of the community at the plot scale because of the lack of plants on bare soil patches; and (3) the plant productivity of the community at the plot scale decreases with the increase in the disturbance intensity of plateau pika because of increased bare soil patches with the enhanced disturbance intensity.

## 2. Results

### 2.1. The Response of Plant Aboveground Biomass at the Quadrat Scale to Plateau Pika Disturbance

**Figure 1:**
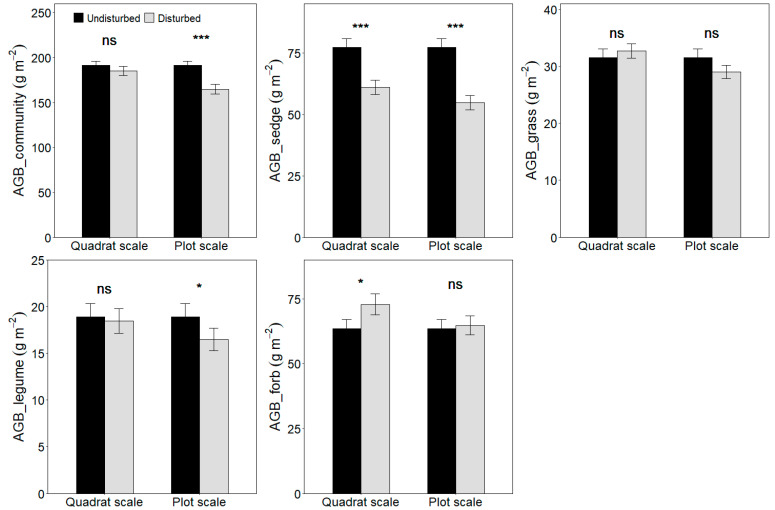
Effect of plateau pika disturbance on plant productivity of community and four functional groups at quadrat and plot scales (Mean ± Std. Error). ABG, aboveground biomass. The statistics were from the LMMs, with the paired plots nested within sites as random factors. For both quadrat and plot scales, ns means no significance between disturbed and undisturbed plots; * means significance between disturbed and undisturbed plots at *p* < 0.05; ***, means significance between disturbed and undisturbed plots at *p* < 0.001.

Considering all sites together, plateau pika disturbance decreased sedge biomass (Figure 1), increased forb biomass, but had no impact on plant community, grass and legume biomass at the quadrat scale.

**Figure 2:**
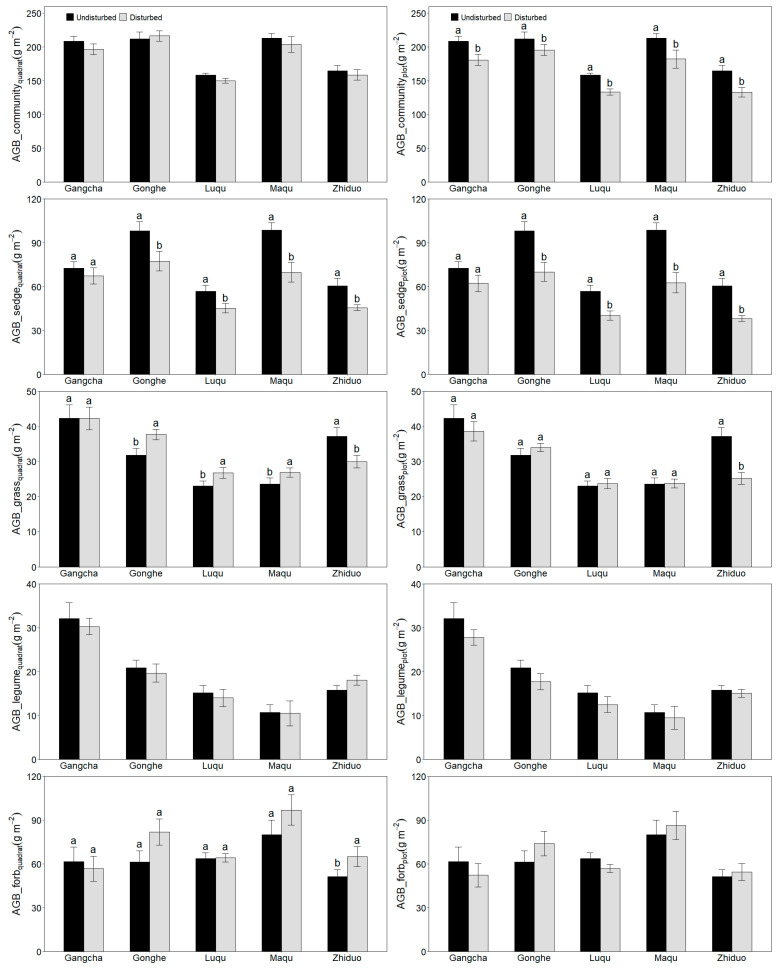
Effect of plateau pika disturbance on plant aboveground biomass at quadrat and plot scales in each site (Mean ± Std. Error). ABG, aboveground biomass. The statistics were from the paired-T test for each site. AGB, aboveground biomass. Different letters indicate a significant difference between the undisturbed plots and disturbed plots at each site (*p* < 0.05).

Considering each site separately, the responses of the plant community and legumes to the disturbance were consistent across the five sites (Figure 2), whereas the responses of sedge, grass and forb to the disturbance were different among the five sites. In Zhiduo, the disturbance decreased sedge and grass biomass, whereas it increased forb biomass at the quadrat scale. In Luqu, Maqu and Gonghe, the disturbance was found to increase grass biomass and decrease sedge biomass, whereas it had no effect on forb biomass. In Gangcha, the plant aboveground biomass of four functional groups was not related to the disturbance.

### 2.2. The Response of Plant Aboveground Biomass at the Plot Scale to Plateau Pika Disturbance

When all sites were considered, plateau pika disturbance decreased plant community, sedge and legume biomass (Figure 1), whereas it had no impact on grass and forb at the plot scale.

When the individual sites were considered, the plant community and forb biomass at the plot scale had similar responses to the disturbance across the five sites (Figure 2). However, the disturbance decreased sedge biomass in Zhiduo, Luqu, Maqu and Gonghe, whereas it did not affect sedge biomass in Gangcha. Additionally, the disturbance decreased grass biomass only in Zhiduo. Although the disturbance decreased legume biomass across all five sites, it had no effect on legume biomass at each site.

### 2.3. Response of Plant Aboveground Biomass to Plateau Pika Disturbance Intensity

**Figure 3:**
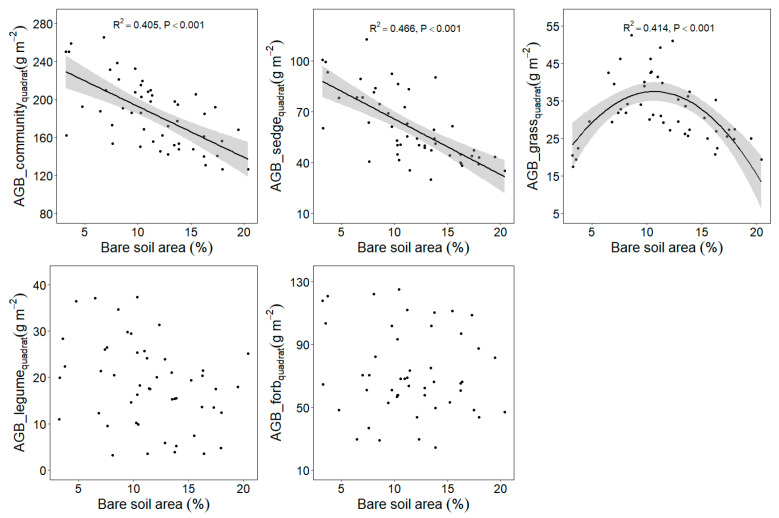
Relationship between plant aboveground biomass at quadrat scale and the bare soil area (%) in the disturbed plots pika across five sites based on a linear model (LM). AGB, aboveground biomass.

**Figure 4:**
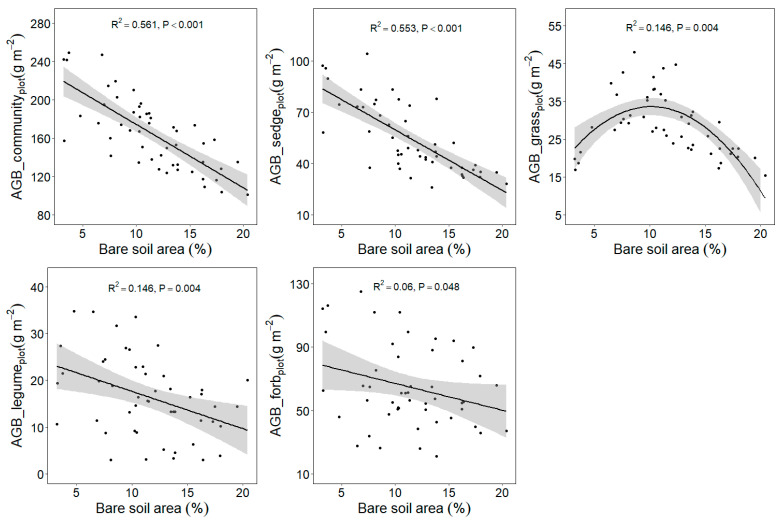
Relationship between plant aboveground biomass at plot scale and the bare soil area (%) in the disturbed plots pika across five sites based on a linear model (LM). AGB, aboveground biomass.

The bare soil area’s average value differed among five sites (Table 1). The relationship between plant aboveground biomass at the quadrat scale and plot scale with plateau pika disturbance intensity are similar (Figure 3 and Figure 4). When the data from five sites were analyzed together, the plant community and sedge biomass showed decreasing trends, whereas the grass biomass first increased and then later decreased at the plot scale, as the plateau pika disturbance intensity increased (Figure 3 and Figure 4). These results presented a general pattern concerning the response of the plant community and four functional groups’ biomass to the disturbance intensity.

**Figure 5:**
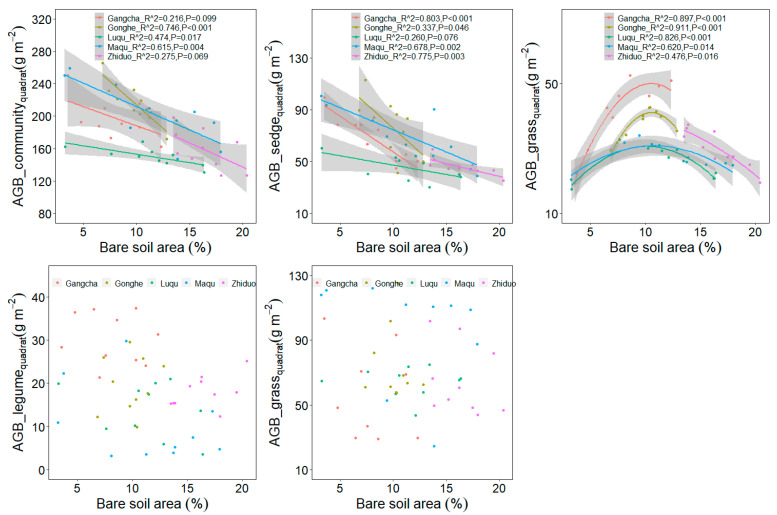
Relationship between plant aboveground biomass at quadrat scale and the bare soil area (%) in each site based on a LM. AGB, aboveground biomass.

**Figure 6:**
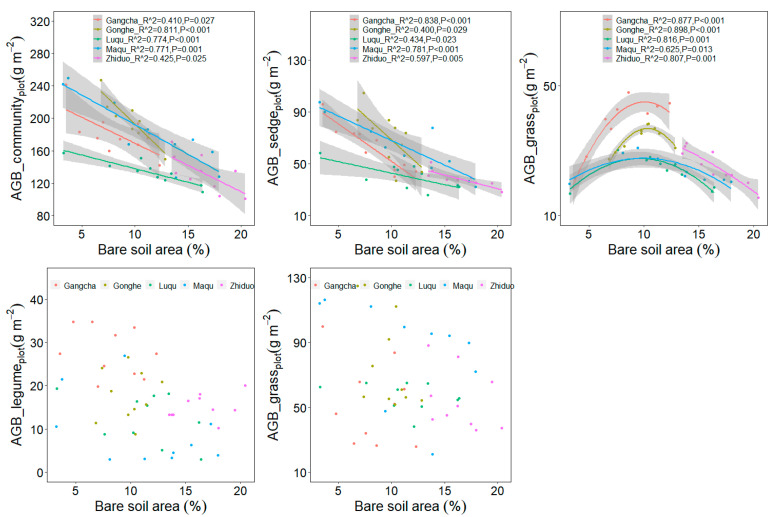
Relationship between plant aboveground biomass at plot scale and the bare soil area (%) in each site based on a LM. AGB, aboveground biomass.

Although the responses of the plant community and sedge biomass to the disturbance intensity were similar to the general pattern at each site (Figure 5 and Figure 6), the response of the grass biomass to the disturbance intensity was different among the five sites. The grass biomass in Gangcha, Luqu, Maqu and Gonghe were in agreement with the general pattern, whereas it showed decreasing trends with the increase of the disturbance intensity in Zhiduo.

## 3. Discussion

Plateau pika disturbance can structure alpine meadows by producing bare soil patches with a lack of plants over a range of spatial scales [11,12], thereby influencing plant productivity. Compared to previous studies [15,18,19,20,21,30], this study examines the effect of plateau pika disturbance on plant aboveground biomass at the quadrat scale (~m^2^) and plot scale (1225 m^2^) simultaneously.

This study shows that plateau pika disturbance had no effect on plant community biomass at the quadrat scale, supporting the first hypothesis, and this is in agreement with some previous studies [20,37], but is not in agreement with other previous studies [18,30]. Similar to this study, both Pang and Guo [20] and Jin et al. [37] placed sampling quadrats only on vegetated surfaces and chose sites as similar as possible in vegetation composition among plots. In contrast, Liu et al. [18] and Sun et al. [30] selected areas with and without disturbance that differed in the dominant species, in which the lower plant community biomass might result from the disturbance or differences in vegetation. In this case, plateau pika can consume minor plants in summer [11], whereas its herbivory can lead to compensatory plant growth that can offset plant consumption [13]. However, this study finds that the disturbance decreases plant community biomass at the plot scale due to the lack of plants on bare soil patches, which confirms the second hypothesis. These findings indicate that plateau pika disturbance can lead to alternative outcomes when the plant aboveground biomass is measured at different scales. Plant aboveground biomass at the plot scale can reflect the difference in plant community biomass between undisturbed and disturbed plots because it considers the effect of bare soil patches induced by plateau pika, and this suggests that the plot scale is better than the quadrat scale for evaluating the effects of the disturbance on plant aboveground biomass. The responses of plant community biomass at two scales to plateau pika disturbance are similar at five sites that range in elevation from 3000 m to 4650 m, where average annual precipitation varies from 290 mm to 800 mm (Table 1). Therefore, the findings of this study are robust even for sites that differ markedly in their environment, which indicates that plot scale is better than quadrat scale to investigate the influence of small burrowing herbivores on the plant aboveground biomass because it not only considers the effect of bare soil patches but also improves the uncertainty of the random sampling method.

This study also shows that plateau pika disturbance has different impacts on the plant aboveground biomass of four functional groups. Plateau pika disturbance decreases sedge biomass at the quadrat and plot scales across five sites, and this is ascribed to the low soil water content of alpine meadows induced by plateau pika disturbance [18,20]; sedge plants, which are generally hygrophytes or mesophytes, allocate more biomass into roots than shoots in relatively dry environments [38]. However, plateau pika disturbance is found to have no impact on sedge biomass at the quadrat scale in Gangcha, where the growth potential of sedge plants in disturbed plots can be maintained because the relatively lower area of bare soil patches (Table 1) in disturbed plots has little effect on soil water content. Besides sedge, grass is a common and frequent plant in alpine meadows at study sites, and it occupies an essential role in the alpine meadow management because of its good quality for livestock [39]. In this case, plateau pika disturbance does not relate to the grass biomass when data from five sites are analyzed together, whereas the effects of plateau pika disturbance on the grass productivity are different among five sites. At the quadrat scale, plateau pika disturbance increases the grass productivity in Luqu, Maqu and Gonghe, whereas it decreases it in Zhiduo. The higher grass productivity at the quadrat scale in Luqu, Maqu and Gonghe may be attributed to two ways: first, the weakened growth potential of sedge plants in the disturbed plots releases competitive ability of grasses [22]; second, the increased soil nitrogen concentration in the disturbed plots [23] is beneficial to grass growth because grasses are nitrophytes [10]. However, in Zhiduo, lower soil nitrogen concentration caused by a relatively larger area of bare soil patches contributes to lower grass biomass. At the plot scale, plateau pika disturbance is found to decrease the grass biomass only in Zhiduo, and this is ascribed to lower grass biomass at the quadrat scale and the larger area of bare soil patches (Table 1). These findings show that the disturbance by plateau pika may improve the grazing quality of alpine meadows through the high grass productivity at some sites, with low areas of bare soil patches, which can provide an example to investigate the effect of small burrowing herbivores on the grazing quality of grassland in other countries or regions.

Additionally, this study shows that the plant community productivity decreases with the increase in the disturbance intensity of plateau pika across five sites, and this is in accordance with the third hypothesis. However, the plant aboveground biomass of four functional groups shows different trends as the disturbance intensity increases. The sedge and legume biomass decrease, whereas the grass biomass shows a hump-shaped curve with the increase in disturbance intensity across the five sites, implying that the optimal disturbance intensity can improve the grazing quality of alpine meadows [39]. However, grass biomass shows site-dependent performance in relation to the disturbance intensity. In Gangcha, Luqu, Maqu and Gonghe, grass biomass is consistent with the general pattern. In Zhiduo, the grass biomass, however, decreases with the increase in the disturbance intensity. The grass biomass peaks at 9–11% bare soil area in Gangcha, Luqu, Maqu and Gonghe. This suggests that the grass productivity may decrease when the bare soil area is over 9–11%. In Zhiduo, the bare soil area is over 13%, which, in turn, supports the results from the other four sites, further validating the general pattern about the effect of the disturbance intensity on the grass biomass. These results suggest that the disturbance seems detrimental to alpine meadows in view of the plant community biomass at the plot scale (Figure 1), whereas intermediate disturbance intensities can improve the grazing quality in alpine meadows (Figure 3 and Figure 4), implying that management of plateau pika needs to consider their disturbance intensity.

## 4. Methods and Materials

### 4.1. Sites Description

Previous studies verified that the plateau pikas live in many habitats with different soil types, topography and microclimates. To identify a general pattern of the presence of plateau pikas influencing plant productivity of alpine meadows across different environments, this study selected five sites in the Qinghai-Tibetan Plateau at Zhiduo, Gonghe, Gangcha, Luqu and Maqu, and these sites are located at 3000 to 4650 m in elevation, with average annual precipitation varying from 290 mm to 800 mm (Table 1). At each site, the alpine meadows are dominated by sedges (*Kobresia humilis* for Gangcha, *Kobresia pygmaea* for Gonghe, *Kobresia pygmaea* for Luqu, *Kobresia pygmaea* for Maqu, and *Carex moorcroftii* for Zhiduo) and are generally divided into warm grazing and cold grazing areas. Cold grazing areas were fenced to avoid grazing from mid-April to early October and were opened to graze yaks (*Bos grunrtiens* L.) and Tibetan sheep (*Ovis aries*) from the middle of October to early April. On the contrary, warm grazing areas were grazed from mid-April to early October, but were fenced to avoid grazing from the middle of October to early April.

### 4.2. Experimental Design

Climate factors regulate plant productivity; thus, to eliminate the microclimates’ effect on plant productivity, this study used a random stratified paired design and ensured paired disturbed-undisturbed plots shared similar soil types, topography and microclimates at each site. Although the plateau zokor (*Myospalax baileyi*), marmot (*Marmota himalayana*) and plateau pika are common burrowing rodents, 10 disturbed plots were selected in areas with only presence of plateau pika at each site, and the distance between the disturbed plots was 3 km to 5 km. Next, a paired and adjacent undisturbed plot, where all small burrowing herbivores were absent, was selected for each disturbed plot at a distance that ranged from 0.5 km to 1 km. For each pair of plots, the disturbed and the undisturbed plots were ensured to be of the same alpine meadow type, with no apparent differences in the vegetation composition. Plateau pikas are territorial and patchily distributed on alpine meadows because they are social mammals that live in family groups, and the sites without plateau pikas may be potential invasion zones. Therefore, undisturbed plots shared the same alpine meadow with disturbed plots and can be easily found because the diffusion of plateau pika from one area to another area is a gradual process. The size of each plot was 35 m × 35 m, approximately equal to the average area of the plateau pika home range [26].

The management measurements were the same for each pair of plots. All plots were fenced to exclude warm grazing by large herbivores (yaks and Tibetan sheep) from mid-April to early October. Fences were opened in the cold grazing season to allow livestock (yaks and Tibetan sheep) grazing from the middle of October to early April.

Generally, many kinds of bare soil patches can be observed in alpine meadows, however, the bare soil patches caused by plateau pika were easily visible and differed from the signs of disturbance caused by factors [12]. This study constricted the bare soil patches to those produced by plateau pika. Each disturbed plot was further divided into the bare soil patches and the vegetated surface. The volume of loose soil generated by wild boar has been used to estimate the disturbance intensity of this ungulate species [40], and the area of bare soil induced by plateau pikas is positively related to the population density of the lagomorph species [23]. Therefore, the total area of bare soil patches in a disturbed plot was used as a proxy for the disturbance intensity of plateau pika [24,25]. Thus, there were 10 paired plots at each site and 100 plots across five sites, consisting of 50 disturbed plots and 50 undisturbed plots.

### 4.3. Field Survey

A field survey was conducted in early August 2017 when the standing biomass and plateau pika disturbance were at their annual peaks. First, the area of each bare soil patch was measured in each disturbed plot, and then the total area of bare soil patches for each disturbed plot was calculated. Each bare soil patch in a disturbed plot was estimated by the following division methods: (a) each bare soil patch was divided into several parts with different shapes, such as square, rectangle, semicircle; (b) sum area of each part was used to estimate the area of each bare soil patch. The average area of bare soil patches induced by plateau pika was different among five 5 sites (Table 1). Second, in each plot, five quadrats with a size of 1 m × 1 m were placed in a W pattern with approximately 8 m of distance between quadrats. In the disturbed plots, all quadrats were placed on vegetated surfaces, avoiding bare soil patches. All aboveground biomass in each 1 m^2^ quadrat was clipped at ground level, carried back to the laboratory. The functional groups of the plant community can reflect the community composition of grassland; therefore, we classified all plant species within the quadrats into the functional groups of sedges, grasses, forbs and legumes based on plant traits [22,41], dried in an oven at 80 °C for 48 h, and then weighed. The sum of the four functional groups per quadrat was used to estimate the aboveground biomass of the plant community at the quadrat scale. The bare soil area of each plot at each site was used to estimate the plant aboveground biomass of that plot [24,25]. Plant aboveground biomass at the plot scale was calculated as following:

AGB_[plot]_ = AGB_[quadrat]_ × (1 − bare soil area (%)) (1)

AGB_[plot]_ is plant aboveground biomass at plot scale, AGB_[quadrat]_ is plant aboveground biomass at quadrat scale. For undisturbed plots, the total area of bare soil patches was considered 0, because this study only considered bare soil patches induced by plateau pika.

### 4.4. Statistical Analyses

All statistical analyses were performed with R 3.5.0 from the R Foundation for Statistical Computing, Vienna, Austria. The normal distribution and homogeneity of variances was tested using Shapiro–Wilk and Bartlett tests. We used Linear Mixed Model (LMM) with the function “lmer” in the lme4 package to examine the effects of plateau pika disturbance on plant aboveground biomass at two scales across the five sites; the paired plots nested within each site were included as a random factor. Furthermore, the data from each of the five sites were analyzed separately to support the general pattern. The paired t-test was used to examine significance in plant aboveground biomass between undisturbed plots and undisturbed plots for each site.

The responses of aboveground biomass at both quadrat scale and plot scale to the percentage of bare soil area were clarified to examine the relationship between plant aboveground biomass and the disturbance intensity. The percentage of bare soil area was considered to be the fixed factor and it was used to construct a regression analysis. The regression curves of plant aboveground biomass at the plot scale/percentage of bare soil patches were obtained using the linear model (LM).

## 5. Conclusions

This study upscales the quadrat scale to the plot scale to examine the effect of plateau pika disturbance on plant aboveground biomass and finds that plateau pika disturbance has no impact on plant community biomass at the quadrat scale but does decrease plant community biomass at the plot scale. Although the plateau pika disturbance seems detrimental to alpine meadows at multiple sites based on the plant community biomass, intermediate plateau pika disturbance intensity [12,23] can improve the grazing quality of alpine meadows due to the higher grass biomass at plot scale. These results suggest that the plot scale is better than the quadrat scale to investigate the influence of plateau pika disturbance on the plant aboveground biomass because it considers the effect of bare soil patches, implying that plant aboveground biomass at the plot scale is a good proxy to examine the relationship between the disturbance by a small burrowing herbivore and grassland sustainability.

## Figures and Tables

**Table 1:** Locations, MAT, MAP, dominant species, main associate species and bare soil bare induced by plateau pika at each of five sites. MAT, mean annual temperature; MAP, mean annual precipitation. Percentage bare soil area of the total plot area (35 m × 35 m) in the disturbed plots was presented as Mean ± Std. Error, and significance was evaluated using one-way ANOVA with the Tukey post hoc test. Different letters denote significant differences at *p* < 0.05.

Location	MAT (°C)	MAP (mm)	Dominant Species	Main Associate Species	Bare Soil Area (%)
Gangcha (99.3°–100.6° E, 36.9°–38° N; 3265 m)	−0.6	370.5	*Kobresia humilis*	*P. pratensis Leontopodium nanum* *Potentilla bifurca*	8.20 ± 0.897 b
Gonghe (99°–101.5° E, 35.5°–37.2° N; 3750 m)	4.1	350	*Kobresia pygmaea*	*E. nutans P. pratensis* *A. obtusiloba*	9.78 ± 0.586 b
Luqu (101.6°–103° E, 34°–34.8° N; 3550 m)	3.3	630	*K. pygmaea*	*Elymus nutans Poa pratensis* *Anemone obtusiloba*	11.41 ±1.234 b
Maqu (100.8°–102.5° E, 33.1°–34.5° N; 3530 m)	2.9	611.9	*K. pygmaea*	*E. nutans P. pratensis* *A. obtusiloba*	11.39 ± 1.651 b
Zhiduo (33°–36.3° E, 89.4°–96.4° N; 4640 m)	−3.8	290.9	*Carex moorcroftii*	*K. pygmaea Poa poophagorum* *L. nanum*	16.41 ± 0.758 a

## Data Availability

Not applicable.
